# Survival of melanoma patients with brain metastases treated with ipilimumab and stereotactic radiosurgery

**DOI:** 10.1002/cam4.315

**Published:** 2014-08-28

**Authors:** Karim Tazi, Amanda Hathaway, Cody Chiuzan, Keisuke Shirai

**Affiliations:** 1 Hematology/Oncology, Medical University of South CarolinaCharleston, South Carolina; 2 Internal Medicine, Medical University of South CarolinaCharleston, South Carolina; 3 Public Health Sciences, Medical University of South CarolinaCharleston, South Carolina

**Keywords:** Brain, ipilimumab, melanoma, metastasis, radiosurgery, stereotactic

## Abstract

Historically, melanoma with brain metastases has a poor prognosis. In this retrospective medical record review, we report the outcome of patients with stage IV melanoma with brain metastases treated with ipilimumab and brain stereotactic radiosurgery (SRS). All patients with metastatic melanoma treated with ipilimumab from June 2010 to September 2012 were identified and stratified by presence (A) or absence (B) of brain metastases at the time of ipilimumab administration. All patients with brain metastases received SRS. Overall survival (OS) was defined as time from the date of stage IV diagnosis and the time of ipilimumab administration to death or last follow-up. Survival curves were estimated using the Kaplan–Meier method, and Cox proportional hazards model was employed to compute the hazard ratios (HR). Results: Five out of 10 patients in Cohort A and 10 out of 21 patients in Cohort B died as of last follow-up. In Cohort A, median number of lesions treated with SRS was 3. Median survivals from date of stage IV for Cohorts A and B were 29.3 and 33.1 months, respectively (HR = 0.93, *P* = 0.896). Median survival from cycle 1 ipilimumab was 16.5 and 24.5 months for Cohort A and B, respectively (HR = 1.05, *P* = 0.931). The 3-year survival rates from the date of cycle one of ipilimumab administration for Cohort A and B were 50% (95% CI: 27–93%) and 39% (95% CI: 19–81%), respectively. Eight of 10 patients in Cohort A maintained a good PS. Survival of patients with melanoma brain metastases treated with ipilimumab combined with SRS may be comparable to patients without brain metastases.

## Introduction

Each year in the United States 76,000 new cases of melanoma are diagnosed and about 9000 patients with melanoma die each year 1. It is the second most frequent invasive cancer in individuals under the age of 39 1 and its incidence has tripled over the past 30 years 2. About 15% of patients will either have metastatic disease at presentation or will develop metastatic disease during the course of their illness 3. Among patients who develop metastatic disease, the prevalence of brain metastases is about 40% and has been shown to be even higher in autopsy series 4. Median survival for stage IV melanoma is <1 year 5. The diagnosis of brain metastases is particularly ominous with a median survival estimated at 4 months from the time of diagnosis 6.

Historically, conventional treatment for brain metastases has been surgical resection and whole brain radiation (WBR) or stereotactic radiosurgery (SRS), but these treatment options have achieved limited success. Patients often present with multiple sites of CNS (central nervous system) involvement and melanoma is particularly radioresistant 7. Ipilimumab is a fully human monoclonal antibody that promotes anti-tumor T Cells by blocking cytotoxic T-lymphocyte-associated antigen 4 (CTLA-4), an immune checkpoint molecule that down-regulates pathways of T-cell activation. It was FDA approved in 2011 and was the first agent to provide a survival benefit based on a phase 3 randomized trial 8. Studies showed a median survival of about 10 months in previously treated patients 8 and around 11 months when used in the first-line setting 9. Only one of these trials included patient with brain metastases and the CNS disease had to be controlled for enrollment 8.

While ipilimumab cannot cross the blood–brain barrier, activated T cells can migrate into the brain and exert an antitumor effect. Evidence of activity of ipilimumab in CNS disease remained anecdotal 10,11, however, recent phase II prospective trials and a retrospective analysis suggested similar activity of ipilimumab in the brain and non-CNS lesions 12–14. In this retrospective analysis, we report that patients with brain metastases, treated with ipilimumab and SRS can have similar outcomes when compared to patients with no brain metastasis. We also show that even extensive, recurrent brain disease could be safely managed with aggressive SRS treatments.

## Methods

Medical records of all patients with stage IV melanoma treated with ipilimumab from June 2010 to September 2012 at the Medical University of South Carolina were reviewed with the approval of the IRB. A total of 31 patients were identified as eligible and included in this analysis. Ten patients had active brain metastases and received SRS before or during ipilimumab treatment and were assigned to Cohort A. The remaining 21 patients did not have brain metastases prior to and by the end of ipilimumab treatment and were assigned to Cohort B. All patients with brain metastases received brain SRS, the exclusive radiation modality for melanoma brain metastasis in our institution, when feasible and when performance status was preserved. Two patients in the study, however, did receive WBR prior to receiving ipilimumab, one in Cohort A and one in Cohort B. We included that patient in Cohort B because he had received WBR adjuvantly for a resected solitary brain metastasis and had no brain recurrence through the end of his ipilimumab therapy. Patients with subsequent disease progression in the brain were evaluated for and treated with SRS when appropriate. Patients with progressive disease in both groups received additional systemic therapy if they were eligible. Data collected from the retrospective review included demographics, lactate dehydrogenase (LDH) levels, performance status at initiation of treatment and throughout the treatment course, number of SRS treatments and number of treated lesions, absolute lymphocyte count (ALC) prior to treatment with ipilimumab and after two cycles, dates of diagnosis and treatments, and patient outcomes. We last updated patients' data in October 2013. The two outcomes considered in this study were OS time, defined as time (months) from stage IV diagnosis to death from any cause and time (months) from the first ipilimumab treatment cycle to death from any cause. The survival rate estimates with the 95% CI were calculated using Kaplan–Meier method. Cox proportional hazards model was employed to generate the hazard ratios and associated p-values.

Information on other prior and subsequent systemic therapies was also recorded. Disease-specific graded prognostic assessment (DS-GPA) was used to estimate predicted survival in patients with brain metastasis in Cohort A.

## Results

**Figure 1:**
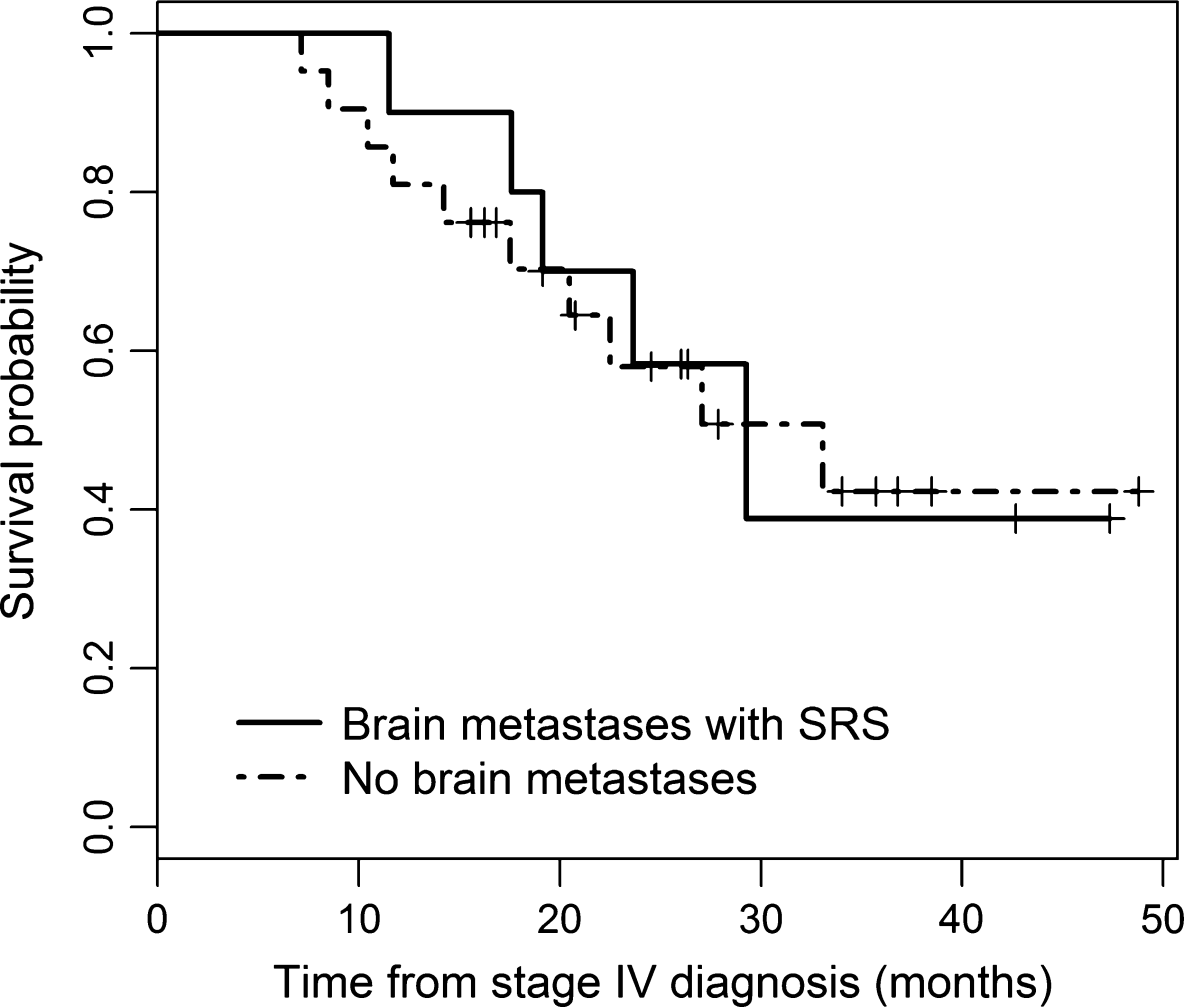
Survival curves from stage IV diagnosis in Cohort A and B, HR = 0.93 (*P* = 0.896).

**Figure 2:**
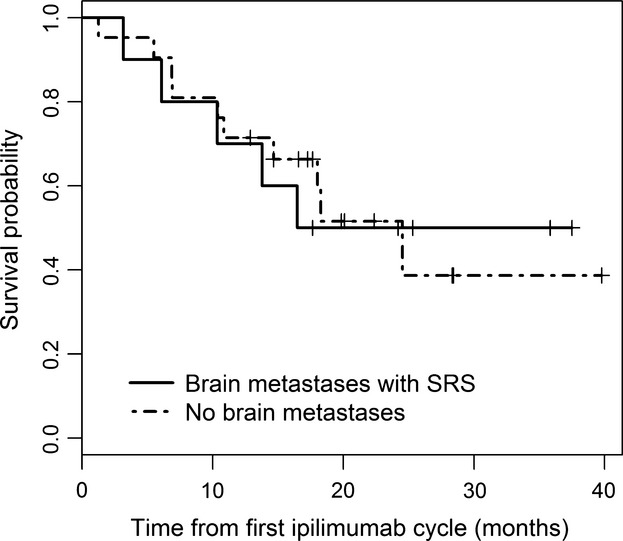
Survival curves from C1 ipilimumab in Cohort A and B, HR = 1.05 (*P* = 0.931).

Patient's characteristics are summarized in Table1. The median age for Cohort A and Cohort B was 66 and 64 years, respectively. The median DS-GPA score in Cohort A was 3 with an estimated mean survival of 9.1 months. Ten patients had brain metastasis and received SRS before or during ipilimumab treatment (Cohort A). Twenty-one patients did not have evidence of active brain metastasis at cycle 1 of ipilimumab and at completion of ipilimumab treatment (Cohort B). Among patients in Cohort B, 7 (33%) developed progression in the brain after the last dose of ipilimumab and six received treatment with brain SRS. One patient died within 6 days of diagnosis of brain metastases. As of last follow-up, 5 (50%) patients in Cohort A died. Four patients died due to progression of melanoma, and one died from aspiration pneumonia secondary to a concurrent head and neck cancer without evidence of melanoma progression. In Cohort B, 10 (48%) patients are deceased, all from progression of melanoma. Median survival from stage IV diagnosis was 29.3 and 33.1 months for Cohorts A and B, respectively (HR = 0.93, *P* = 0.896) (Fig.1 and Table2). The median number of ipilimumab cycles was four in both cohorts with the range of 2–11 cycles in Cohort A and a range of 1–8 cycles in Cohort B. Two patients in Cohort A and five patients in Cohort B received more than one course (four cycles) of ipilimumab as they had either an objective response or a prolonged progression-free interval with a good Eastern Cooperative Oncology Group Performance Status (PS). Median survival from cycle 1 ipilimumab was 16.5 and 24.5 months for Cohorts A and B, respectively (HR = 1.05, *P* = 0.931) (Fig.2 and Table3). The estimated 3-year survival rates from the date of cycle one of ipilimumab administration for Cohorts A and B were 50% (95% CI: 27–93%) and 39% (95% CI: 19–81%), respectively (Table3). Eight out of 10 patients in Cohort A maintained an ECOG performance status of 0–1 during the period of active therapy and at 1-year follow-up despite brain metastases. Two (20%) patients in Cohort A and 3 (14%) patients in Cohort B had abnormal LDH at cycle 1 ipilimumab. Adverse events are summarized in Table4. Only one (10%) patient in Cohort A experienced more than grade 2 toxicities (grade 3 diarrhea). Five (24%) patients in Cohort B experienced more than grade 2 toxicities. Three had grade 3 diarrhea and one had grade 3 hypopituitarism. Three (30%) patients in Cohort A and 10 (48%) patients in Cohort B received additional systemic therapy after ipilimumab (Table1).

**Table 1:** Patient characteristics

Characteristic	Cohort A (*N* = 10)	Cohort B (*N* = 21)	*P*-value*
Age	65.5 (41–81)	64 (43–89)	0.949
Stage Iva	0	0	N/A
Stage IVb	0	9	N/A
Stage IVc	10	12	N/A
No. of sites of metastasis	3 (2–5)	3 (1–5)	0.275
DS-GPA	3 (1–4)	N/A	N/A
ECOG PS at stage IV diagnosis	1 (0–2)	1 (0–1)	0.517
ECOG PS at last follow-up	1 (0–2)	1 (0–3)	0.360
LDH at stage IV diagnosis	151 (117–268)	153 (121–416)	0.370
LDH at last clinic follow-up	204 (101–946)	178 (123–995)	0.724
No. of cycles of ipilimumab	4 (2–11)	4 (1–8)	0.211
No. of SRS lesions treated	2 (1–59)	0 (0–20)	0.008
No. of SRS sessions	2 (1–5)	0 (0–5)	0.003
No. of lesions treated per session	2 (1–32)	1 (0–12)	0.017
			*P*-value*
Male	5 (50%)	16 (76%)	0.222
BRAF+	6 (60%)	8 (38%)	0.441
Deceased at last follow-up	5 (50%)	10 (47.6%)	0.999
Receive ≥4 cycles of ipilimumab	8 (80%)	14 (66.7%)	0.677
Received ipilimumab as first-line treatment	6 (60%)	10 (47.6%)	0.704
Received treatment after ipilimumab	3 (30%)	10 (47.6%)	0.452
Experienced ipilimumab side effects	3 (30%)	14 (66.7%)	0.121
Brain metastasis	10 (100%)	7 (33%)	0.0004
Radiation other than SRS	3 (30%)	14 (66.7%)	0.120

Values are represented as median (range) and No. (%) of patients. *P*-values were generated by Wilcoxon Rank-Sum test^*^ and Fisher exact test^*^^*^ at a significance level of 0.05. SRS, stereotactic radiosurgery.

**Table 2:** Overall survival—time from stage IV diagnosis to the last follow-up (months)

Group	*N*	No. of deaths	Median survival (95% CI)	Survival (95% CI) 1 year	Survival (95% CI) 2 years	Survival (95% CI) 3 years
Brain metastases with SRS (Cohort A)	10	5	29.3 (19.1–NA)	0.90 (0.71–1.00)	0.58 (0.27–0.90)	0.39 (0.01–0.76)
No brain metastases (Cohort B)	21	10	33.1 (20.5–NA)	0.81 (0.64–0.98)	0.58 (0.35–0.81)	0.42 (0.17–0.67)
Total	31	15	29.3 (20.5–NA)	0.84 (0.71–0.97)	0.57 (0.39–0.76)	0.41 (0.20–0.62)

SRS, stereotactic radiosurgery.

**Table 3:** Overall survival—time from first ipilimumab cycle to the last follow-up (months)

Group	*N*	No. of deaths	Median Survival (95% CI)	Survival (95% CI) 1 year	Survival (95% CI) 2 years	Survival (95% CI) 3 years
Brain metastases with SRS (Cohort A)	10	5	16.5 (10.4–NA)	0.70 (0.47–1.00)	0.50 (0.27–0.93)	0.50 (0.27–0.93)
No brain metastases (Cohort B)	21	10	24.5 (14.7–NA)	0.71 (0.55–0.94)	0.52 (0.32–0.82)	0.39 (0.19–0.81)
Total	31	15	24.5 (14.7–NA)	0.71 (0.57–0.89)	0.51 (0.36–0.74)	0.44 (0.27–0.71)

SRS, stereotactic radiosurgery.

**Table 4:** Significant toxicities

	Cohort A (*N* = 10)		Cohort B (*N* = 21)	
Adverse event	Any grade	Grade 3 or 4	Any grade	Grade 3 or 4
GI (diarrhea, colitis)	2 (20%)	1 (10%)	(14.3%)	3 (14.3%)
Skin (rash, pruritus)	0 (0%)	0 (0%)	2 (9.5%)	1 (4.8%)
Liver dysfunction	1 (10%)	0 (0%)	0 (0%)	0 (0%)
Pituitary insufficiency	1 (10%)	0 (0%)	7 (33.3%)	1 (4.8%)

### Ipilimumab first line versus second line

Five (50%) patients in Cohort A and 9 (43%) patients in Cohort B received prior systemic therapy for metastatic disease. In Cohort A, one received vemurafenib, one received high-dose Interleukin-2, two received temozolomide, and two were on clinical trials. For these patients, median survival from stage IV diagnosis was 23.6 months and 13.8 months from cycle one of ipilimumab. In Cohort B one patient received vemurafenib, six received temozolomide, and two were on clinical trials prior to ipilimumab initiation. Median survival was not reached from stage IV diagnosis and 18 months from cycle one of ipilimumab.

In patients who received ipilimumab as first-line treatment, median survival from stage IV diagnosis and cycle one ipilimumab was 29.3 months and not reached respectively in Cohort A and 27.1 and 24.5 months, respectively in Cohort B.

Two patients in Cohort A received more than one course of ipilimumab. Their median survival from cycle one of ipilimumab had not been reached yet. In Cohort B, five patients received more than one course of ipilimumab. Their median survival was 24.5 months from cycle one of ipilimumab.

### SRS data

The median number of SRS treatments in Cohort A was two with a range of one to five treatment sessions and the median total number of lesions treated was two with a range of 1–59. In Cohort B, seven patients developed brain metastases after ipilimumab treatment. Median survival from cycle one of ipilimumab in these patients was 18 months, 95% CI (6.9–NA). Median survival from cycle one of ipilimumab in patients who did not develop brain metastases was not reached. In Cohort A, one patient received SRS to a total of 59 lesions in five sessions as of last follow-up. That particular patient's tumor harbored a sensitizing BRAF mutation and received vemurafenib after progression on ipilimumab therapy. His PS was two as last follow-up. His systemic disease was extensive as well with metastases to the brain, kidney, adrenals, and lungs. He died from his disease in hospice after surviving 571 days from his initial stage IV diagnosis and 494 days from his first ipilimumab treatment. Three other patients received “aggressive” SRS treatment: one had eight lesions treated (four sessions) and two others had five lesions treated (two sessions). Survival in these three patients was 750 days, 698 days, and 1323 days from stage IV diagnosis and 724 days, 406 days, and 985 days from C1 ipilimumab, respectively.

### BRAF mutation status

Six patients in Cohort A (60%) and eight patients in Cohort B (39%) had tumors harboring BRAF mutation. One patient in Cohort A received a BRAF inhibitor prior to initiation of ipilimumab and survived 17.4 months after stage IV diagnosis. Three patients in Cohort A received a BRAF inhibitor after ipilimumab treatment with median survival of 29.3 months, with one patient still alive at time of analysis. The other two patients were progression free and did not receive BRAF inhibitor treatment. In Cohort B, one patient received a BRAF inhibitor prior to treatment with ipilimumab and survival was 7.1 months from stage IV diagnosis. In Cohort B, four patients received BRAF inhibitors after ipilimumab treatment with a median survival of 11.7 months (two patients were still alive as of last follow-up). The remaining three patients with tumors harboring BRAF mutation in Cohort B were progression free at the time of analysis and were not treated with BRAF inhibitors.

## Discussion

This retrospective analysis confirms prior observations of prolonged survival of patients treated with the combination of ipilimumab therapy and brain SRS. Shoukat et al. 15 examined patient with brains metastases undergoing SRS with and without the addition of ipilimumab and reported an improvement in median OS with combination of SRS with ipilimumab (28.3 vs. 6.8 months). Knisely et al. 14 also reported a survival improvement in patients receiving ipilimumab supported with SRS control of brain metastases. In our cohort, we were able to compare two groups with similar baseline characteristics that were treated with ipilimumab based on the same criteria within a single center. We show that patients with brain metastases not only exceeded their predicted survival based on their DS-GPA but also had similar outcomes when compared with patients without brain metastasis at presentation. It is noteworthy that among all patients with metastatic melanoma who presented to our institution during the observation period, only two were not eligible for immune therapy. Four patients received “aggressive” SRS salvage and achieved a remarkable survival time. This shows the feasibility of treating recurrently a large number of CNS lesions without significantly impacting patient's quality of life.

We have been surprised by the prolonged estimated survivals noted in Cohort B as one would expect median survivals in that cohort to be comparable to the one found in Robert et al. 9 study where median survival in the dacarbazine + ipilimumab group was 11.2 months. This is likely a reflection of the small sample size and the fact that, many patients have received treatment with BRAF-targeted therapy during the observation period.

We hypothesize that the addition of SRS may enhance ipilimumab-induced immune response perhaps through an enhanced antigen presentation within the tumor stroma 16. We have not noted significant side effects despite treatment of a large number of brain lesions and patients preserved a good performance status throughout the treatment period. Rate of grade 3–4 adverse events was in line to what was reported in registrational trials in both groups. Our analysis is limited by the sample size and its retrospective nature. However, being a single institution with a homogenous population treated in a relatively uniform manner, it would allow a noteworthy comparison in our two respective groups.

## Conclusion

This retrospective analysis suggests that patients with brain metastases can achieve an impressive survival with the combination of ipilimumab and SRS. Furthermore, we show the relative safety and feasibility of aggressive SRS treatment of brain metastases. Further prospective randomized studies are warranted to determine optimal timing of SRS therapy in combination with ipilimumab and the benefit of incorporating radiation early on in treating metastatic melanoma. Finally, based on our and others observation that patients with melanoma with brain metastasis seem to derive comparable benefit from modern systemic therapy as patient without brain metastasis, CNS disease should not be an exclusion criteria in future clinical trials.

## Conflict of Interest

Keisuke Shirai: Consultant, Bristol-Myers Squibb.
